# Cardiovascular fitness of forensic psychiatric patients: a longitudinal clinical study

**DOI:** 10.1186/s12991-026-00680-3

**Published:** 2026-07-01

**Authors:** Anja Fernqvist, Peter Andiné, Roland Thomeé, Alessio Degl’Innocenti, Thomas Nilsson, Annelie Gutke

**Affiliations:** 1 Centre for Ethics, Law and Mental Health, Department of Psychiatry and Neurochemistry, Institute of Neuroscience and Physiology, The Sahlgrenska Academy, University of Gothenburg, Gothenburg, Sweden; 2 Department of Forensic Psychiatry, National Board of Forensic Medicine, Gothenburg, Sweden; 3 Department of Forensic Psychiatry, Sahlgrenska University Hospital, Region Västra Götaland, Gothenburg, Sweden; 4 Unit of Physiotherapy, Department of Health and Rehabilitation, Institute of Neuroscience and Physiology, Sahlgrenska Academy, University of Gothenburg, Gothenburg, Sweden; 5 Regionhälsan, Region of Västra Götaland, Gothenburg, Sweden

**Keywords:** Forensic psychiatry, Physical activity, Physiotherapy, Cardiovascular fitness

## Abstract

**Background:**

Forensic psychiatric patients have a reduced life expectancy, largely due to cardiovascular diseases. Low maximal oxygen uptake is a risk factor for cardiovascular diseases in the general population and may also be essential to the cardiovascular risk among forensic psychiatric patients. The purpose of this study was to verify previous results of very low estimated maximal oxygen uptake levels in a larger group of forensic psychiatric patients and investigate the impact of the length of inpatient forensic psychiatric care on estimated maximal oxygen uptake. Further, we examined the extent to which BMI, level of physical activity, and smoking status were associated with estimated maximal oxygen uptake.

**Methods:**

We evaluated estimated levels of maximal oxygen uptake based on clinical testing of 115 forensic psychiatric patients and the development of these levels during inpatient care for those who underwent retesting (two tests, n = 66, three tests, n = 30).

**Results:**

Mean estimated levels of maximal oxygen uptake were remarkably low, confirming previous findings. Levels correlated negatively with higher body mass index, lower physical activity, and older age; however, no change was observed during forensic psychiatric inpatient care. Estimated physical activity levels tended to increase between tests one and two, but decreased significantly between tests two and three.

**Conclusions:**

This Swedish forensic psychiatric cohort had very low cardiovascular fitness. The strongest associated factors were a high BMI and a low level of physical activity. These results make essential contributions to the planning of future studies on treatment strategies aimed at improving metabolic health in forensic psychiatric patients. The importance of aerobic exercise, as well as means to encourage patients to reach and maintain recommended levels of physical activity, needs to be further explored.

**Supplementary Information:**

The online version contains supplementary material available at 10.1186/s12991-026-00680-3.

## Background

Patients at forensic mental health services in Sweden have been sentenced to forensic psychiatric care. They have all been judged to have a severe mental disorder. In most cases, this is equal to a severe mental illness, typically a psychotic disorder (e.g., schizophrenia). Lengths of stay are long [1] and characterised by psychiatric treatment and rehabilitation, aiming to reintegrate patients into society. Severe mental illness entails an increased risk of several physical illnesses, which contribute to increased mortality rates and considerably shorter expected life span compared with the general population in both psychiatric [2–4] and forensic [5–7] patients.

Physical and psychiatric health are dependent on and affect one another, and the concept of integral brain health was recently suggested to form a base for collaboration between psychiatrists and neurologists [8]. Brain health is defined by the World Health Organization (WHO) as the optimal functioning of sensory, motor, cognitive and emotional systems [9]. Different aspects of lifestyle, including physical activity and exercise, affect brain functions. Physical activity and exercise are essential risk modifiers for several relevant somatic diseases, like metabolic syndrome and cardiovascular diseases [10, 11], known to contribute substantially to the shorter life expectancy of patients with severe mental illness. Further, physical exercise also enhances cognitive functions [12, 13] and reduces psychiatric symptoms such as depression, anxiety and positive as well as negative symptoms in schizophrenia [14, 15]. It might also improve the effect of cognitive training in patients with schizophrenia [16]. Measures to improve cardiovascular fitness may improve cognitive function, the outcome of rehabilitation, and reduce lengths of stay in forensic psychiatric care, thereby reducing involuntary measures for the patients and costs for forensic mental health services [17].

In a previous study at the present study site, a forensic mental health service in Sweden, 28 patients with a mean age of 33 years had very low estimated maximal oxygen uptake capacity (VO₂max) levels [18]. Low maximal oxygen uptake equals a low physical performance level [19] and is a risk factor for cardiovascular disorders in the general population [20, 21]. This may play an essential role in the cardiovascular risk among forensic psychiatric patients. At the present study site, recommendations for physical exercise are based on physical performance tests within existing clinical assessment and treatment routines. However, the effect of these routines is unclear. Data from the Swedish National Forensic Psychiatry Register [22, 23] suggest that patients' body mass index (BMI, kg/m^2^), a measure that correlates negatively with VO₂max in groups of patients with schizophrenia [24], has not improved since 2008, when the register was initiated. 

Implementing health care for somatic conditions in forensic psychiatric patient groups has proven to be challenging [25]. One possible explanation is a discrepancy between self-rated and clinically assessed health, with patients often underestimating their somatic disease burden [26]. There is a need to supplement treatment strategies aiming to reduce the risk of metabolic and cardiovascular diseases within this patient group. 

Incorporating treatment measures that improve physical performance into forensic psychiatric rehabilitation could have a significant potential for improving both physical and psychiatric health in this patient group. To design effective treatments, it is necessary to explore potential causes of low VO₂max. Admission to forensic psychiatric inpatient care in Sweden carries several circumstances that can negatively affect the patients’ levels of estimated VO₂max. A large proportion will be prescribed antipsychotic medication, regardless of diagnosis, indicating prevalent off-label prescription [22]. Treatment with antipsychotic medication can cause several side effects, possibly reducing VO₂max capacity. One common side effect is increased body weight, thereby increasing BMI [27, 28]. Another is sedation due to anticholinergic effects [29], contributing to physical inactivity along with restrictive safety measures in a forensic psychiatric setting. In addition, smoking is more common among patients with schizophrenia compared to the general population [30] and affects VO₂max capacity negatively [31].

More extensive studies are needed to confirm the low levels of estimated VO₂max in the forensic psychiatric patient group and to generate hypotheses for adequate treatment options that can at least reduce the risk of metabolic and cardiovascular diseases. 

To address these needs, the current study aimed to examine test results for estimated VO₂max in a larger group of forensic psychiatric patients and the impact of the length of inpatient forensic psychiatric care on estimated VO₂max. A secondary aim was to investigate whether BMI, level of physical activity, and smoking status were associated with estimated VO₂max levels. We hypothesised that mean estimated VO₂max levels would be lower compared to the general population and decrease with an increased length of stay in inpatient forensic mental health services, even after accounting for the impact of BMI, level of physical activity, and smoking status. 

## Materials & methods

### Participants

The study cohort comprised patients from four rehabilitation wards at a forensic mental health service in Sweden who completed physical performance testing as part of regular clinical examinations and treatment between September 1, 2014, and June 30, 2022. In Sweden, a sentence to forensic psychiatric care can be combined with special court supervision if the risk of recidivism into serious crime has been assessed to be elevated, which usually leads to longer lengths of stay. The study cohort included patients with and without special court supervision. All data were collected retrospectively from existing medical records.

**Fig. 1:**
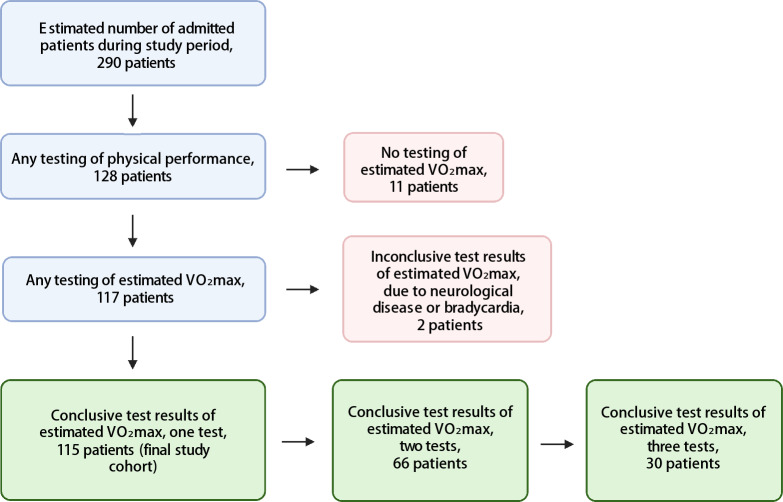
Flowchart

During the study period, all patients admitted to forensic psychiatric care at the study site were offered the opportunity to assess their cardiovascular fitness in accordance with existing clinical routines, thereby becoming eligible for the study. However, we were not able to obtain the exact number of admitted patients from available registers since the study site receives not only newly convicted but also patients transferred from other forensic mental health services in Sweden and abroad, and patients reconvicted during ongoing forensic psychiatric outpatient care. Each year, approximately 100 patients receive inpatient care at the study site, while about 25 patients leave, and another 25 enter the hospital. Thus, the estimated number of patients admitted during the study period was 290 (see Fig. 1) We had no information on the reasons why patients refrained from cardiovascular fitness tests, as this was not recorded in the existing medical records.

Inclusion criteria for the present study were: (1) patients admitted for forensic psychiatric care at Sahlgrenska University Hospital, Sweden, and (2) who had completed one or more tests of physical performance of the outcome variable estimated VO₂max during forensic psychiatric rehabilitation between September 1, 2014, and June 30, 2022. The exclusion criterion was: patients with inconclusive results from testing the outcome variable (estimated VO₂max). Inclusion and exclusion criteria were assessed during an initial review of the test results, resulting in 115 patients being included in the final study cohort (see Fig. 1, Flowchart). All testing of estimated VO₂max was performed using the Åstrand-Rhyming test [32, 33], a valid, reliable, and clinically useful test with age-adjusted reference values as described below.

### Procedure

A doctoral student, also a clinical physiotherapist (HB), identified patients who had completed physical performance tests using the outcome variable estimated VO₂max during the specified period. A research nurse (ÅJ) and another doctoral student, also a senior consultant (AF), collected and registered all data from clinical medical records in a study protocol and evaluated the inclusion and exclusion criteria. In a few cases where the information was unclear, ambiguous or difficult to interpret, a senior researcher and senior consultant (PA) reassessed the collected data. Final decisions on how to register the data point in question were made in consensus after a joint discussion. Two patients were excluded due to inconclusive estimated VO₂max results (see Fig. 1, Flowchart).

### Materials

At the study site, patients are offered physical testing to estimate VO₂max capacity using the Åstrand-Rhyming submaximal cycle ergometer test as part of regular clinical examination and treatment. In the test, the steady-state heart rate is measured during the last minute of a six-minute submaximal cycling session at a constant work rate. VO₂max is estimated using a nomogram that includes steady-state heart rate, workload, and sex. Reference values are corrected for age and have recently been updated for the Swedish general population, intended for use in clinical evaluation [34]. ‘Estimated VO₂max’, expressed in ml O₂ per minute per kg body weight as recorded in medical records during the specified study period, was the study’s outcome variable.

We considered the length of inpatient forensic psychiatric care from admission to when the Åstrand-Rhyming test was performed as our primary explanatory variable. We recorded the admission date for inpatient forensic psychiatric care and the date of each test to calculate the variable ‘Length of stay’ in months.

To explore the secondary aim of this study, BMI and level of physical activity were recorded, provided it was dated within one month of each test occasion. Both BMI and information on physical activity were assessed by staff during recurring health check-ups, as part of regular clinical routines, and noted in medical records. In these check-ups, the recommended level of physical activity was considered achieved if the assessing staff estimated that the participants’ physical activity met or exceeded 150 min of moderate-intensity, heart rate-increasing activity per week. The variable ‘Assessed level of physical activity’ was recorded dichotomously as fulfilled or not, while the variable ‘BMI’ was recorded as a continuous variable in kg/m^2^. The variable ‘Smoking status’ was assessed once per participant, and dichotomously as ‘yes’ or ‘no’. A participant was considered a habitual smoker if continuous smoking habits were described at the time of testing or, if repeated tests were made, during the majority of the period covering the tests.

For descriptive purposes, we recorded: each participant’s age at testing, sex, psychiatric diagnoses and whether the care was combined with a special court supervision. We used data from clinical psychological tests of intellectual functioning to record whether each participant had an impairment of intellectual ability or not. Furthermore, we recorded somatic diagnoses and all standing prescriptions of medications at each test, including pro re nata medications given on three or more occasions within a week preceding each test. Psychiatric diagnoses and intellectual functioning were classified according to the Diagnostic and Statistical Manual of Mental Disorders, Fifth Edition (DSM-5) [35], and somatic diagnoses were classified according to the International Classification of Diseases, Eleventh Revision (ICD-11) [36]. Medications were classified according to the Anatomical Therapeutic Chemical Classification (ATC) [37].

Subgroups of participants had completed repeated testing. Outcome and exploratory variables were recorded for up to three subsequent estimated VO₂max tests. The number of participants who had completed more than three tests was deemed too few for statistical analysis. Descriptive variables were recorded for all test occasions.

To increase our study’s validity and clinical relevance, we compared our study cohort with the overall forensic psychiatric population [38], assessing the ecological validity of our study cohort by comparing participant characteristics to corresponding measures in the annual report from the Swedish National Forensic Psychiatric Register, RättspsyK, during the corresponding period [23].

### Statistics

Statistical analyses were performed in IBM SPSS version 29.0.2.0. We calculated the variables Length of stay and Time between tests in months. We created a variable to represent the difference in estimated VO₂max between tests, and from that, a binned variable where the difference was either equal to or less than zero (unchanged or reduced estimated VO₂max) or more than zero (increased estimated VO₂max). Furthermore, we created a categorical variable for BMI corresponding to underweight, normal weight, overweight, and obesity classes I, II and III, respectively, as defined by the WHO [39]. Descriptive analyses were performed for all variables. Since reference values for VO₂max are sex-specific, we calculated sex-specific mean values of VO₂max in the study cohort for comparative purposes.

Primary exploratory analyses (comparisons of central tendencies and regression analyses) included the outcome variable Estimated VO₂max and explanatory variables Length of stay, BMI, Assessed level of physical activity, and Smoking status to evaluate the primary and secondary aims. Evaluation of continuous variables revealed that the outcome, Estimated VO₂max, and the predictor BMI could be treated as normally distributed variables with no extreme values present. Length of stay was skewed due to a few participants having very long inpatient care periods.

Measures of frequencies and central tendency (mean, median) with respective dispersion measures (percentages, standard deviations, and interquartile ranges) were calculated for continuous variables. Comparisons of central tendencies between test occasions in the same participant were made with paired t-tests for the normally distributed variables, Estimated VO₂max and BMI. For the dichotomous Assessed level of physical activity, we used McNemar’s Test for paired analyses. Correlation was evaluated using Pearson’s correlation coefficient, provided the assumptions of linearity and normal distribution of the data were met. Otherwise, we used Spearman’s rank correlation coefficient. Since a large proportion of participants performed their first test within the first six months of their care period, we investigated whether this subgroup was especially vulnerable to a decrease in physical performance with increasing length of stay. The clinical guidelines included refraining from taking prescribed beta-blockers, known to likely impair test results due to their pharmacological effect on restricting heart rate, on the day of the Åstrand-Rhyming test; however, compliance with this recommendation could not be verified. Thus, we decided to perform sensitivity analyses excluding participants prescribed beta-blockers.

We used linear regression to evaluate the explanatory and adjusting variables to the dependent variable separately. In the next step, we used a multiple linear regression model, excluding cases pairwise to eliminate participants with missing data points, resulting in 90 participants with complete data for all variables. A post hoc power analysis, assuming a medium effect, alpha 0.05, and four explanatory variables, yielded a power of 82%. We checked the residuals for normality and confirmed that the model's conditions were met, as evidenced by the adequacy of the Q-Q plots. Variance inflation factor values ranged from 1.000 to 1.184, indicating a low degree of multicollinearity between the explanatory variables. Regression analyses were adjusted for age, since VO₂max levels are known to vary with age in the general population.

## Results

### Descriptives

Participant characteristics for test 1 – 3 are presented in Tables 1 and 2. To summarise significant findings, the proportion of men was around 97% across all three test occasions. At the times of testing, 6.7–7.8% of participants were prescribed beta-blockers, and 6.1–6.7% of participants had musculoskeletal diseases, which may have impaired their ability to perform the test. Regarding prevalent neurological diseases, none were likely to significantly impact mobility. The mean age was similar at each test occasion, revealing that older participants did not conduct repeated testing to the same extent as younger participants. BMI mean values were close to the cutoff for obesity. Of the 104 participants with a registered BMI at the first test, 43 had a BMI corresponding to overweight (BMI 25 – 29.9), and another 34 to obesity (BMI ≥ 30 kg/m^2^), as categorised by the WHO. Participants who performed four tests (n = 6) were all male. The mean age at baseline was slightly higher (32.17 years) than in the entire study cohort, and the majority had a BMI corresponding to overweight rather than obesity (mean BMI 27.03 kg/m^2^). Otherwise, baseline characteristics were consistent with those of the entire study cohort. A majority had a psychosis diagnosis (5/6,83.3%), were prescribed antipsychotic medication (5/6, 83.3%), were habitual smokers (4/6, 66.7%), and their care was combined with special court supervision (5/6, 83.3%). Regarding the level of physical activity, there were some missing data, and individual data are not reported. Only two of these participants performed five tests.

**Table 1:** Participant characteristics, per number of repeated Åstrand-Rhyming tests

	Test number 1	Test number 2	Test number 3
	n = 115	n = 66	n = 30
Forensic psychiatric care with special court supervision,			
% (n)	89.6 (103)	90.9 (60)	93.3 (28)
Sex, prop of men,			
% (n)	96.5 (111)	97 (64)	96.7 (29)
Age in years,			
Median (IQR) Mean (SD)	30.00 (12.00) 31.85 (9.07)	31.00 (10.00) 31.97 (8.48)	31.50 (11.00) 31.5 (7.55)
Age in years, men,			
Median (IQR) Mean (SD)	30.00 (11.00) 31.61 (8.99)	31.00 (9.75) 31.77 (8.27)	31.00 (10.00) 30.83 (6.70)
Estimated VO₂max, ml O₂/min/kg,			
Median (IQR) Mean (SD)	24.00 (10.10) 25.24 (7.42)	24.20 (10.60) 25.53 (8.21)	25.40 (6.05) 25.73 (5.98)
Length of stay in months at test,			
Median (IQR) Mean (SD)	4.00 (12.00) 19.83 (39.42)	13.00 (11.25) 26.15 (39.27)	20.50 (19.00) 35.87 (44.71)
Time since previous test in months,			
Median (IQR) Mean (SD) Maximum—minimum	-	9.00 (6.00) 11.88 (9.71) 3.00 – 41.00	7.50 (5.50) 12.27 (11.47) 3.00 – 58.00
Habitual smoker,			
% (n) Missing, % (n)	74.8 (86) 3.5 (4)	75.8 (50) 4.5 (3)	73.3 (22) 6.7 (2)
Assessed to achieve recommended physical activity level,			
% (n) Missing, % (n)	22.6 (26) 11.3 (13)	37.9 (25) 9.1 (6)	26.7 (8) 20.0 (6)
BMI, in kg/m^2^,			
Median (IQR) Mean (SD) Missing, % (n)	27.60 (6.50) 28.45 (4.96) 10.4 (12)	28.75 (6.27) 29.17 (4.49) 15.1 (10)	30.00 (6.25) 29.78 (5.03) 13.3 (4)
BMI, WHO categories,			
Underweight, % (n) Normal weight, % (n) Overweight, % (n) Obesity, % (n) Missing, % (n)	- 22.6 (26) 37.4 (43) 29.5 (34) 10.4 (12)	- 12.1 (8) 37.9 (25) 34.7 (23) 15.1 (10)	3.3 (1) 6.7 (2) 33.3 (10) 43.3 (13) 13.3 (4)
Psychotic and/or bipolar disorders,			
% (n)	78.3 (90)	74.2 (49)	80.0 (24)
Antipsychotic medication,			
% (n)	92.2 (106)	87.9 (58)	80.0 (24)
Any somatic diagnosis,			
% (n)	57.4 (66)	63.6 (42)	70.0 (21)
Somatic diagnosis relevant to physical performance levels*,			
% (n)	40.9 (47)	50.0 (33)	53.3 (16)
Musculoskeletal disease,			
% (n)	6.1 (7)	6.1 (4)	6.7 (2)
Medicated with beta-blockade at test,			
% (n)	7.8 (9)	6.1 (4)	6.7 (2)
Borderline intellectual functioning or intellectual disability,			
% (n) Missing, % (n)	30.4 (35) 29.6 (34)	25.8 (17) 24.2 (16)	26.7 (8) 16.7 (5)

n = number of participants, IQR = interquartile range, SD = standard deviation, VO₂max = maximal oxygen uptake capacity, BMI = body mass index, WHO = World Health Organization

^*^Obesity, metabolic syndrome, diabetes mellitus, hypertension, hyperlipidaemia, anaemia, chronic heart disease, tachycardias, obstructive lung disease and musculoskeletal diseases, including rheumatoid and neurological diseases affecting muscular function

**Table 2:** Participant characteristics, group that performed three Åstrand-Rhyming tests

	Test number 1	Test number 2	Test number 3
	n = 30	n = 30	n = 30
Forensic psychiatric care with special court supervision,			
% (n)	93.3 (28)	93.3 (28)	93.3 (28)
Sex, prop of men,			
% (n)	96.7 (29)	96.7 (29)	96.7 (29)
Age in years,			
Median (IQR) Mean (SD)	28.00 (11) 29.60 (7.69)	29.50 (11) 30.43 (7.59)	31.50 (11.00) 31.5 (7.55)
Age in years, men,			
Median (IQR) Mean (SD)	28.00 (11.00) 29.50 (7.12)	28.00 (9.50) 29.67 (6.65)	31.00 (10.00) 30.83 (6.70)
Estimated VO₂max, ml O₂/min/kg,			
Median (IQR) Mean (SD)	25.35 (8.45) 25.82 (6.04)	24.60 (9.50) 25.43 (5.83)	25.40 (6.05) 25.73 (5.98)
Length of stay in months at test,			
Median (IQR) Mean (SD)	4.00 (4.00) 12.50 (36.95)	11.00 (8.00) 12.50 (36.95)	20.50 (19.00) 35.87 (44.71)
Time since previous test in months,			
Median (IQR) Mean (SD) Maximum—minimum		7.50 (6.00) 11.10 (9.40) 3.00 – 39.00	7.50 (5.50) 12.27 (11.47) 3.00 – 58.00
Habitual smoker,			
% (n) Missing, % (n)	73.3 (22) 6.7 (2)	73.3 (22) 6.7 (2)	73.3 (22) 6.7 (2)
Assessed to achieve recommended physical activity level,			
% (n) Missing, % (n)	16.7 (5) 20.0 (6)	46.7 (14) 10.0 (3)	26.7 (8) 20.0 (6)
BMI, in kg/m^2^,			
Median (IQR) Mean (SD) Missing, % (n)	28.20 (4.45) 28.13 (3.42) 13.3 (4)	29.04 (6.00) 28.35 (4.31) 6.7 (2)	30.00 (6.25) 29.78 (5.03) 13.3 (4)
BMI, WHO categories,			
Underweight, % (n) Normal weight, % (n) Overweight, % (n) Obesity, % (n) Missing, % (n)	- 16.7 (5) 43.3 (13) 26.7 (8) 13.3 (4)	- 10.0 (3) 46.7 (14) 36.7 (11) 6.7 (2)	3.3 (1) 6.7 (2) 33.3 (10) 43.3 (13) 13.3 (4)
Psychotic and/or bipolar disorders,			
% (n)	80.0 (24)	80.0 (24)	80.0 (24)
Antipsychotic medication,			
% (n)	93.3 (28)	90.0 (27)	80.0 (24)
Any somatic diagnosis,			
% (n)	56.7 (17)	70.0 (21)	70.0 (21)
Somatic diagnosis relevant to physical performance levels*,			
% (n)	40.0 (12)	53.3 (16)	53.3 (16)
Musculoskeletal disease,			
% (n)	10.0 (3)	6.7 (2)	6.7 (2)
Medicated with beta-blockade at test,			
% (n)	10.0 (3)	10.0 (3)	6.7 (2)
Borderline intellectual functioning or intellectual disability,			
% (n) Missing, % (n)	26.7 (8) 16.7 (5)	26.7 (8) 16.7 (5)	26.7 (8) 16.7 (5)

n = number of participants, IQR = interquartile range, SD = standard deviation, VO₂max = maximal oxygen uptake capacity, BMI = body mass index, WHO = World Health Organization

^*^Obesity, metabolic syndrome, diabetes mellitus, hypertension, hyperlipidaemia, anaemia, chronic heart disease, tachycardias, obstructive lung disease and musculoskeletal diseases, including rheumatoid and neurological diseases affecting muscular function

At baseline, the proportion of participants in the entire cohort (n = 115) with a diagnosis of psychosis was the same as that reported in the Swedish National Forensic Psychiatry Register RättspsyK during the corresponding period [23]. Our study also included some differences compared to what is seen in RättspsyK since participants were somewhat more often male (approx. 97% vs. 85%), younger than 40 years old (approx. 78% vs. 66%), and in forensic psychiatric care combined with special court supervision (approx. 91% vs. 77%).

### Estimated VO_2_max levels

**Fig. 2:**
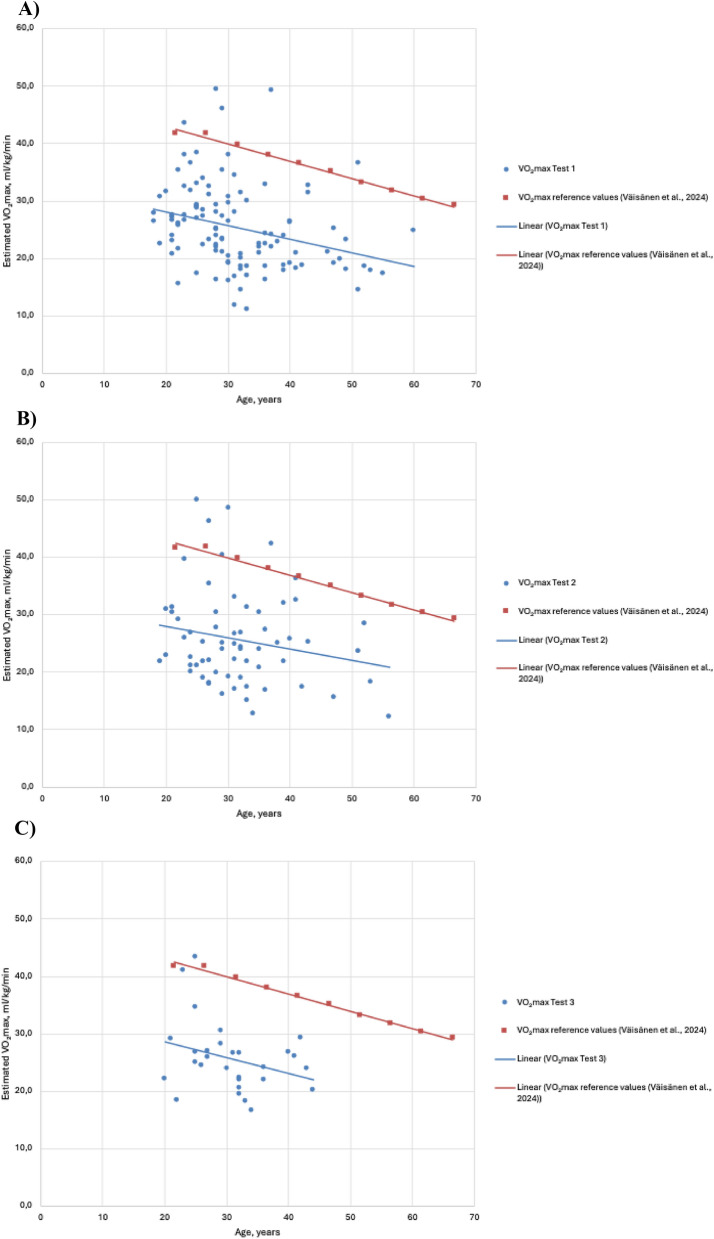
Estimated VO₂max levels. (A) Male participants, estimated VO₂max vs Age. Test 1, n = 111. (B) Male participants, VO₂max vs Age. Test 2, n = 64. (C) Male participants, VO₂max vs Age. Test 3, n = 29.

Figures 2a, b, c show results for the male study cohort at Tests 1, 2, and 3, respectively, compared to gender-specific and age-adjusted reference values from a Swedish normal population, developed for use in clinical evaluations as described in the Materials & Methods Section [27]. VO₂max values of the male study cohort, with mean ages of approximately 32 years, were lower than average values, comparable to somewhat low values for 65–69-year-old men [34]. Female participants were deemed too few for subgroup analysis. Results for the subgroup with a length of stay of up to six months to their first test were similar. As indicated in Table 1, mean and median levels of estimated VO₂max were stable across tests. Mean estimated VO₂max levels for the small group of participants (n = 6) performing four tests also corresponded to very low values for their age group (24.58 – 26.58 – 28.12 – 27.57 ml O₂/min/kg). All of them performed their first test within three to seven months after admission, and the mean time between test occasions ranged from seven to eleven months.

### Comparisons between tests

The study cohort was reduced by about half for each repeated Åstrand-Rhyming test, with 66 participants performing two tests and 30 participants performing three tests (See Fig. 1). There were no significant changes in estimated levels of VO₂max in any of the groups between tests, as shown in Table 3. When examining the binned estimated VO₂max variable, only one significant connection was found. Achieving a recommended level of physical activity indicated a higher proportion of a positive change in estimated VO₂max between Tests 1 and 3 (p = 0.032), even though the expected count was small.

**Table 3:** Comparisons of central tendencies, subgroups that performed two or three tests

Subgroup	Performed Test 1 and 2		Performed Test 2 and 3		Performed Test 1 and 3	
Total n	66		30		30	
	Test 1	Test 2	Test 2	Test 3	Test 1	Test 3
Estimated VO₂max						
Mean, ml O₂/min/kg (SD)	25.78 (7.66)	25.53 (8.21)	25.43 (5.83)	25.73 (5.98)	25.82 (6.04)	25.73 (5.98)
Mean difference, ml O₂/min/kg (SD, 95% CI), two-sided *p*	-0.25 (6.20, -1.77 – 1.27), 0.744		0.30 (5.64, -1.80 – 2.41), 0.770		-0.08 (6.64, -2.56 – 2.40), 0.946	
n	66		30		30	
BMI						
Mean, kg/m² (SD)	28.19 (4.48)	29.01 (4.32)	29.31 (4.48)	29.73 (5.12)	28.52 (3.51)	29.96 (5.01)
Mean difference, kg/m² (SD, 95% CI), two-sided *p*	0.82 (2.54, -0.10 – -1.53), 0.026*		-0.43 (2.35, -1.40 – 0.54), 0.371		1.44 (3.30, -0.03 – 2.90), 0.054	
n	51		25		22	
Assessed to achieve recommended physical activity level, % (n)	19.2 (10)	34.6 (18)	45.5 (10)	27.3 (6)	15.8 (3)	31.6 (6)
Change in proportion achieved, two-sided *p*	0.057		0.031*		0.375	
n	52		22		19	
Habitual smoker, % (n)	79.4 (50)		78.6 (22)		78.6 (22)	
n	63		28		28	

n=number of participants with valid values, IQR=interquartile range, SD=standard deviation, CI=confidence interval, VO₂max=maximal oxygen uptake capacity, BMI=body mass index, *p*=probability value

The proportion of participants who achieved a recommended level of physical activity tended to increase between Tests 1 and 2 (22.6% to 37.9%, p = 0.057) and decrease between Tests 2 and 3 (37.9% to 26.7%, p = 0.031) (Table 3). Mean BMI values increased 0.82 kg/m^2^ (p = 0.026) between Tests 1 and 2, with no difference observed between Tests 2 and 3. In the subgroup with up to six months to first test, the mean BMI increase between the first and second tests was 1.08 kg/m^2^ (p = 0.005).

### Regressions

The results from linear regression analysis for each explanatory and adjusting variable at Test 1 are presented in Table 4. In addition, correlation coefficients are presented in Table 5 (supplements). When analysed separately, BMI made the most considerable contribution to the variance of estimated VO₂max, with a coefficient of determination (adjusted R-squared, ARS) of 0.34. Assessed level of physical activity contributed but with a low ARS of 0.10. Length of stay alone did not explain any variance in estimated VO₂max.

**Table 4:** Linear regressions, Test 1

Dependent variable: Estimated VO₂max, ml O₂/min/kg body weight						Correlations	
Predictor	n	Unstandardized regression coefficient B (ml O₂/min/kg body weight)	Probability value *p*	95% CI for B	Explained variance, adjusted R^2^	Correlation coefficient ρ	Probability value *p*
Length of stay	115	-0.025	0.151	-0.060 – 0.009	0.009	-0.060 (S)	0.527
Constant		25.739	< 0.001***	24.212 – 27.267			
BMI	103	-0.859	< 0.001***	-1.091 – -0.626	0.341	-0.589 (P)	< 0.001***
Constant		49.387	< 0.001***	42.676 – 56.099			
Physically Active	102	5.307	< 0.001***	2.336 – 8.278	0.103	0.313 (S)	< 0.001***
Constant		23.332	< 0.001***	21.832 – 24.831			
Habitual smoker	111	-3.573	0.034*	-6.875 – -0.270	0.032	-0.167 (S)	0.081
Constant		28.040	< 0.001***	25.133 – 30.947			
Age	115	-0.210	0.006**		0.058	-0.351 (S)	< 0.001***
Constant		31.922					

n = number of participants with valid values, VO₂max = maximal oxygen uptake capacity, CI = confidence interval, R^2^ = coefficient of determination, (S) = Spearman’s, (P) = Pearson’s. Significant probability values in bold text

The results of multiple regression analysis are presented in Table 5. Tests of interaction between outcome variables, including the adjusting variable Age, did not yield any significant effects. In multiple regression, the explanatory degree was somewhat higher than for BMI alone with an ARS value of 0.37. Significant contributors were BMI (p < 0.001) and level of physical activity (p = 0.021). In our model, estimated VO₂max decreased by 0.715 ml O₂/minute/kg body weight with each kg/m^2^ increase in BMI. Physical activity below the recommended level decreased the estimated VO₂max by 3.547 ml O₂/minute/kg compared to participants who achieved the recommended level of physical activity. Exploratory sensitivity analyses excluding participants prescribed beta-blockers, those with musculoskeletal diseases, or both, resulted in little or no change in the significance levels of B-coefficients or ARS. In multiple regression analyses conducted on the smaller groups that completed two and three tests, results indicated that BMI and level of physical activity remained significant explanatory variables. (Table 6, supplements).

**Table 5:** Multiple regression, Test 1

Dependent variable:Estimated VO₂max, ml O₂/min/kg body weight. n = 90					
Population	Predictor	Unstandardized regression coefficient B (ml O₂/min/kg body weight)	95% CI for B	Probability value *p*	Explained variance, adjusted R2
VO₂max, all	Constant / Intercept	48.247	41.096 – 55.398	< 0.001***	
	Length of stay	- 0.021	- 0.061 – 0.019	0.303	
	**BMI**	**- 0.726**	**- 0.970 – -0.483**	< 0.001***	
	**Physically inactive**	**- 3.547**	**- 6.536 – -0.557**	0.021*	
	Non-smoker	1.710	- 1.254 – 4.673	0.255	
					0.369
VO₂max, all, age-adjusted	Constant / Intercept	49.800	42.122 – 57.478	< 0.001***	
	Length of stay	-0.016	-0.057 – 0.026	0.457	
	**BMI**	**-0.705**	**-0.951 – -0.458**	< 0.001***	
	**Physically inactive**	**-3.495**	**-6.483 – -0.507**	0.022*	
	Non-smoker	1.817	-1.150 – 4.783	0.227	
	Age	-0.072	-0.203 – 0.059	0.276	
					0.370

n = number of participants with valid values, VO₂max = maximal oxygen uptake capacity, CI = confidence interval, R^2^ = coefficient of determination. Significant probability values in bold text

## Discussion

The findings in this study, based on medical records from 115 inpatients at a forensic psychiatric hospital in Sweden, confirmed earlier findings of very low levels of cardiorespiratory fitness among forensic psychiatric patients [18]. The mean estimated VO₂max levels of the male study group, with a mean age of 32 years, were lower than the average estimated VO₂max levels of men aged 65–69 years. Levels of estimated VO₂max neither worsened nor improved when tests were repeated during inpatient care. However, the subgroups of study participants who underwent repeated testing were considerably smaller and decreased in size with each test. Furthermore, the length of stay did not correlate with or explain any variance in estimated VO₂max, and the results were the same when examining a subgroup with a shorter length of stay at their first test occasion. This implies that patients in forensic psychiatric care not only experience severe mental illness before admission but also very poor cardiorespiratory fitness. Contributing factors might be a sedentary lifestyle associated with previous episodes of severe mental illness, including previous restrictions of mobility and side-effects of medical treatments at psychiatric institutions and/or correctional services.

A somewhat positive finding was that cardiorespiratory fitness did not deteriorate during inpatient compulsory care, despite safety measures restricting the possibilities for patients to be physically active. Still, estimated VO₂max levels are very low, and healthcare should aim to improve this critical risk factor for common physical diseases. There is already convincing evidence that mental and physical health are closely entangled, especially in the most severely mentally ill [40, 41]. Although this circumstance has been known for decades, the physical health in this group is not improving [41, 42]. Clinical routines for follow-up of physical health appear to be challenging to implement in forensic psychiatric settings, at least in terms of adequately treating and improving patients' physical health status. Notably, in this study cohort, 28 out of 34 participants whose BMI met the criteria for obesity did not have a registered diagnosis of obesity in their medical records. With many forensic psychiatric patients being overweight or obese, there is a risk for normalisation and underestimation of, for example, cardiovascular risks, even among healthcare professionals. As expected, BMI was inversely correlated with estimated VO₂max in the study cohort, accounting for 34% of the variance in VO₂max. As 34 out of 104 participants with registered BMI at the first test were obese, and another 43 were overweight, this reflects an area of great concern, as previously shown in Swedish forensic psychiatric populations [43].

Achieving a recommended level of physical activity showed a positive correlation to estimated VO₂max levels. Although the explanatory degree of VO₂max variance was low, it suggests that physical activity levels matter in this patient group and may be a potential target for future interventions aimed at improving cardiorespiratory fitness. Contrary to expectations, smoking status did not turn out to be an important explanatory factor of VO₂max in this cohort. Smoking status can be difficult to assess [44, 45], which is further discussed in the section Strengths and limitations.

The causal relations between BMI, cardiorespiratory fitness, and higher mortality are currently being scientifically discussed. Cardiorespiratory fitness in the general population has proved to be strongly associated with all-cause and cardiovascular mortality, whereas the elevated mortality risk associated with BMI disappeared when cardiorespiratory fitness was considered [46]. However, recent findings also suggest that a causal effect of cardiorespiratory fitness on mortality cannot be adopted to the extent previously assumed without further research, due to the potential confounding effects of socioeconomic, genetic, and behavioural factors [47]. Still, with current knowledge, we consider it clear that overweight, obesity, and low cardiorespiratory fitness are essential targets for interventions aiming to improve the physical health and ultimately reduce the high mortality rates from physical diseases such as cardiovascular disease, diabetes, and cancer in patients with severe mental illness.

With pressing needs for treatment of severe mental symptoms affecting everyday functioning and safety for both patients and clinical staff, treatment of psychiatric symptoms is, and must be, highly prioritised. The use of antipsychotic medication such as clozapine and olanzapine, with a disadvantageous profile with regard to metabolic and anticholinergic side effects [27], is common in Swedish forensic psychiatry [23]. This might reflect patient group characteristics, e.g., a high prevalence of treatment-resistant psychotic symptoms. However, it also raises concerns about existing clinical routines for evaluation of benefits versus drawbacks of psychotropic medication that would motivate switching to medication with a more favourable metabolic and anticholinergic profile. There is a lack of evidence-based treatment regimes in Swedish forensic psychiatry, and a need for further research to cover this gap. Given the close relationship between mental and physical health, prioritising improved physical health can also benefit the overall outcomes of forensic psychiatric rehabilitation. By ameliorating psychiatric symptoms such as anxiety, depression, or positive and negative psychotic symptoms in patients where episodes of deterioration and pronounced psychiatric symptoms often have proven to result in aggression, preventive health care measures such as physical activity can theoretically improve even the safety at forensic psychiatric wards. Implementing treatment regimens that include physical activity and exercise can be challenging. Participants performing three repeated tests of estimated VO₂max achieved, to a higher degree, recommended levels of physical activity as assessed by health professionals. In this group, there was a trend towards increasing levels of physical activity at test occasion number two, followed by a decrease at test occasion number three. Although the numbers are small, this aligns with clinical observations of difficulties in maintaining motivation for treatments, including physical exercise programs, during prolonged periods of inpatient care where patients have little opportunity to influence the length of care. Thus, treatment programs aiming to increase levels of physical activity need to include adequate supporting measures to enhance motivation.

### Strengths and limitations

The major strength of this study lies in the size of the cohort, with as many as 115 participants contributing to the evaluation of cardiovascular fitness, which, to our knowledge, is unique in a forensic psychiatric setting. Since we do not have the exact numbers on how many patients at the hospital were eligible to test their cardiovascular fitness during the study period, but refrained from doing so, the study still adheres to selection bias. Each year, approximately 100 patients receive inpatient care at the study site, while about 25 patients leave, and another 25 enter the hospital. This means that roughly 290 patients were admitted for inpatient care during the study period, out of which 115 were tested with the Åstrand-Rhyming test at least once. Although a selection bias could not be ruled out, we successfully enrolled 40% of the patients.

Further, the study is retrospective and based on information from medical records, which introduces limitations. A baseline test at admission would be ideal to avoid the confounding effects of treatment interventions on the factors studied. In clinical practice, the first test occasion is often postponed due to the patient experiencing severe psychiatric symptoms that might take a relatively long time to treat. In addition, we do not have information on patients’ reasons for not participating in cardiovascular fitness testing. Possible reasons for patients to abstain from Åstrand-Rhyming tests are unwillingness or inability to participate due to psychiatric and/or physical deterioration, or lack of motivation. Patients could also have been deemed unfit to perform an Åstrand-Rhyming test by their treating psychiatrist due to safety or health-related reasons. The higher levels of physical activity in the group that performed three repeated Åstrand-Rhyming tests suggest a possible type II error due to selection bias. Other conceivable reasons for patients not to participate are transfer to another forensic psychiatric hospital or discharge from forensic psychiatric inpatient care. Still, as reported in the descriptive results section, our participant group resembles the Swedish forensic psychiatric population as described in the register RättspsyK. If anything, the described differences suggest that our main finding of low cardiovascular fitness corresponds to even lower levels in the entire forensic psychiatric population.

Another limitation is the difficulty in categorising data on smoking status, which, contrary to expectations, was not associated with estimated VO₂max levels in our analysis. This is a common methodological difficulty. Even though participants were not described as habitual smokers during the period of physical performance testing, they may well have been during other parts of their lives, still affecting levels of VO₂max. Thus, smoking status is a variable likely to be misclassified.

Taken together, our findings of very low cardiovascular fitness in a Swedish forensic psychiatric cohort confirm earlier preliminary findings. Low cardiovascular fitness constitutes an essential part of the multiple burdens that contribute to poor brain health in this patient group. Since lengths of stay in forensic psychiatric care in Sweden are long-lasting, it can be recommended to offer aerobic exercise with adequate motivating support, which could have the potential to reduce a significant risk factor for cardiovascular disease in forensic psychiatric patients.

## Conclusions

The main conclusion of this study is that a clinical cohort of Swedish forensic psychiatric patients had very low cardiovascular fitness throughout the course of forensic psychiatric care, and that the strongest factors associated with this result were a high BMI and a low level of physical activity. The results of this study make essential contributions to decision-making when planning interventions to ameliorate this vital risk factor for cardiovascular disease in forensic psychiatric patients. Further, improved cardiovascular fitness may improve brain health and thereby enhance cognitive functions and reduce psychiatric symptoms, potentially contributing to shorter lengths of stay in forensic psychiatric care. The importance of aerobic exercise, as well as means to encourage patients to reach and maintain recommended levels of physical activity, needs to be further explored.

## Supplementary Information

Additional file 1.

Additional file 2.

## Data Availability

The data that support the findings of this study are available from the corresponding author upon reasonable request.

## Abbreviations

VO₂max: Maximal oxygen uptake capacity
BMI: Body mass index
DSM-5: The Diagnostic and Statistical Manual of Mental Disorders, Fifth Edition
ICD-11: The International Classification of Diseases, Eleventh Revision
ATC: The Anatomical Therapeutic Chemical Classification
WHO: World Health Organization
ARS: Adjusted R-squared
RättspsyK: The Swedish National Forensic Psychiatric Register
ALF: Swedish Agreement on PhysicianEducation and Clinical Research (Avtal om Läkarutbildning och Forskning)
HB: Henrik Bergman
ÅJ: Åsa Johansson
AF: Anja Fernqvist
PA: Peter Andiné
