# Supplementary material for: Cardiovascular fitness of forensic psychiatric patients: a longitudinal clinical study
Source: Ann Gen Psychiatry. 2026 Jul 1;25:63. doi: 10.1186/s12991-026-00680-3 (PMC13386802; doi:10.1186/s12991-026-00680-3)
Supplement: Supplementary file 1 — Additional file 1. [file 12991_2026_680_MOESM1_ESM.docx]

| Table 5. Correlations Test 1. Correlation coefficients ρ, (probability value *p*), n of limiting variable | | | | | | |
| --- | --- | --- | --- | --- | --- | --- |
|  | 1.  Estimated VO₂max | 2.  Length of stay | 3.  Smoker | 4.  BMI | 3.  Physically active | 4.  Age |
| 1. Estimated VO₂max | - | -0.060, (0.527), 115 | -0.167  (0.081)  111 | -0.589  **(<0.001)**  103 | 0.313  **(0.001)**  102 | -0.351  **(>0.001)**  115 |
| 2. Length of stay | -0.060, (0.527), 115 | - | -0.078  (0.415)  111 | 0.063  (0.528)  103 | 0.029  (0.773)  102 | 0.084  (0.373)  115 |
| 3. Smoker | -0.167  (0.081)  111 | -0.078  (0.415)  111 | - | 0.068  (0.502)  99 | -0.210  **(0.037)**  99 | -0.041  (0.668)  111 |
| 4. BMI | -0.589  **(<0.001)**  103 | 0.063  (0.528)  103 | 0.068  (0.502)  99 | - | -0.134  (0.200)  93 | 0.282  **(0.004)**  103 |
| 3. Physically active | 0.313  **(0.001)**  102 | 0.029  (0.773)  102 | -0.210  **(0.037)**  99 | -0.134  (0.200)  93 | - | -0.090  (0.367)  102 |
| 4. Age | -0.351  **(>0.001)**  115 | 0.084  (0.373)  115 | -0.041  (0.668)  111 | 0.282  **(0.004)**  103 | -0.090  (0.367)  102 | - |
| Pearson’s ρ for VO₂max vs BMI, all other correlations Spearman’s ρ, VO₂max=maximal oxygen uptake capacity in ml O₂/kg/min. Significant probability values in bold text. | | | | | | |
