# Supplementary material for: Cardiovascular fitness of forensic psychiatric patients: a longitudinal clinical study
Source: Ann Gen Psychiatry. 2026 Jul 1;25:63. doi: 10.1186/s12991-026-00680-3 (PMC13386802; doi:10.1186/s12991-026-00680-3)
Supplement: Supplementary file 2 — Additional file 2. [file 12991_2026_680_MOESM2_ESM.docx]

| Table 6. Multiple regression. Sensitivity and subgroup analyses. Dependent variable: Estimated VO₂max, ml O₂/min/kg body weight. | | | | | |
| --- | --- | --- | --- | --- | --- |
| Population | Predictor | Unstandardized regression coefficient B (ml O₂/min/kg body weight) | 95% CI for B | Probability value  *p* | Explained Variance, Adjusted R² |
| VO₂max Test 1 without beta-blockers, n = 83 | Constant / Intercept | 48.238 | 40.910 – 55.565 | **<0.001***** |  |
|  | Length of stay | -0.017 | -0.060 – 0.026 | 0.433 |  |
|  | BMI | -0.715 | -0.963 – -0.466 | **<0.001***** |  |
|  | Physically inactive | -4.129 | -7.300 – -0.958 | **0.011*** |  |
|  | Non-smoker | 2.047 | -1.037 – 5.132 | 0.190 |  |
|  |  |  |  |  | 0.375 |
|  |  |  |  |  |  |
| VO₂max Test 1 without musculoskeletal diseases, n = 84 | Constant / Intercept | 48.968 | 41.410 – 56.526 | **<0.001***** |  |
|  | Length of stay | -0.017 | -0.059 – 0.024 | 0.414 |  |
|  | BMI | -0.752 | -1.007 – -0.496 | **<0.001***** |  |
|  | Physically inactive | -3.873 | -6.872 – -0.874 | **0.012*** |  |
|  | Non-smoker | 1.488 | -1.554 – 4.531 | 0.333 |  |
|  |  |  |  |  | 0.381 |
|  |  |  |  |  |  |
| VO₂max Test 1 without beta-blockers and musculoskeletal diseases, n = 78 | Constant / Intercept | 49.498 | 41.682 – 57.315 | **<0.001***** |  |
|  | Length of stay | -0.013 | -0.058 – 0.032 | 0.561 |  |
|  | BMI | -0.757 | -1.021 – -0.494 | **<0.001***** |  |
|  | Physically inactive | -4.314 | -7.531 – -1.097 | **0.009**** |  |
|  | Non-smoker | 1.713 | -1.500 – 4.926 | 0.291 |  |
|  |  |  |  |  | 0.384 |
|  |  |  |  |  |  |
| VO₂max Test 1, up to six months length of stay at first test, n = 59 | Constant / Intercept | 52.311 | 41.082 – 63.539 | **<0.001***** |  |
|  | Length of stay | 0.049 | -1-021 – 1.119 | 0.602 |  |
|  | BMI | -0.814 | -1.180 – -0.448 | **<0.001***** |  |
|  | Physically inactive | -5.700 | -9.463 – -1.937 | **0.004**** |  |
|  | Non-smoker | 0.931 | -2.959 – 4.821 | 0.161 |  |
|  |  |  |  |  | 0.329 |
|  |  |  |  |  |  |
| n=number of participants with valid values, VO₂max=maximal oxygen uptake capacity, CI=confidence interval, R^2^=coefficient of determination. Significant probability values in bold text. | | | | | |
